# Analysis of the spatio-temporal evolution of sustainable land use in China under the carbon emission trading scheme: A measurement idea based on the DID model

**DOI:** 10.1371/journal.pone.0285688

**Published:** 2023-05-22

**Authors:** Yingjuan Ma, Haoyuan Feng, Yanjun Meng, Longfei Yue

**Affiliations:** 1 School of Economics and Management, Lanzhou University of Arts and Science, Lanzhou, China; 2 School of Ecological and Environmental Sciences, East China Normal University, Shanghai, China; 3 School of Business, Hechi University, Guangxi, China; 4 School of Physical Education, Sichuan University, Chengdu, China; Northeastern University (Shenyang China), CHINA

## Abstract

Sustainable development is the theme of world economic development in the 21st century. As a key part of sustainable development, sustainable land use (SLU) encompasses economic development and environmentally friendly and social progress. In recent decades, China has formulated many environmental regulatory policies to achieve sustainable development and "carbon peaking and carbon neutrality (double-carbon)" goals, among which the carbon emission trading scheme (CETS) is the most representative and provides valuable research. In this paper, we aimed to reflect the spatio-temporal evolution of SLU in China under the influence of environmental regulatory policies through an indicator measurement strategy based on the DID estimation method. The study conclusions are as follows: (1) The CETS can effectively improve SLU from the perspectives of economic development and environmentally friendly progress, and the impact has primarily been in the pilot areas. And, its effectiveness is closely linked to local locational factors. (2) With respect to the dimension of economic development, the CETS has not changed the provincial distribution patterns of SLU; rather, it continues to remain "high to low, east to west". However, regarding the environmentally friendly progress dimension, the CETS has significantly changed the provincial distribution patterns of SLU, which are characterized by spatial agglomeration with urban agglomerations such as the Pearl River Delta (PRD) and the Yangtze River Delta (YRD) as the core. (3) The screening results of the SLU indicators based on economic development showed that the CETS primarily improved the innovation capacities of pilot regions, and the impacts on economic levels were relatively small. Similarly, the screening results of the SLU indicators based on environmentally friendly progress showed that the CETS had primarily acted on reducing pollution emission intensity and strengthening greening construction, revealing only short-term effects on improving energy use efficiency. Based on the above, this paper explored the meaning and role of the CETS in more detail, with a view to providing insight into the implementation and formulation of environmental regulation policies.

## 1. Introduction

Sustainable development is an important theme for the development of countries around the globe in the 21st century. The concept of sustainable development originated at the World Conservation Strategy in 1980, which aimed to create a general public awareness of the limited capacity of natural resources and ecosystems to support human beings in their pursuit of economic development and enjoyment of natural wealth and that the needs of future generations must be taken into account. “Sustainable development” was formally introduced in 1987 in the report Our Common Future. The Rio Declaration emphasized that environmental protection was the key to sustainable development and called for all countries to do their part to promote development together. For the individual, modern man has an ever-increasing need for a suitable living environment [1]. The climate of the living environment is influenced not only by natural factors, but also by human economic activities [2]. Therefore, the search for sustainable development has become an important element in the course of human civilisation. Sustainable development involves nature, the environment, society, the economy, science and technology, politics and many other fields. Researchers have discussed the importance of sustainable development from their own fields in a multidimensional way, including ecological and environmental perspectives [3–5], human health [6–8], and economic development [9–12].

Sustainable land use (SLU) is the exploration of sustainable development at the level of land use. Our Common Future defined SLU resources as minimizing damage and degradation to land resources on which human survival depends, maintaining a constant or increasing capital stock, and pursuing maximum economic benefits while maintaining and improving the productive conditions and environmental base of land resources. The idea of SLU was formally recognized at the International Symposium on Land Use Systems in 1990. In 1993, the Food and Agriculture Organization of the United Nations published the Outline for Sustainable Land Use Assessment, which emphasized that SLU should combine technology, policy and activities aimed at simultaneously caring for socio-economic principles and the environment. It can be observed that SLU does not emphasize the nature of "land" alone, but rather, it emphasizes the natural (ecological, etc.), social and economic natures of land as a vehicle. Based on these definitions, scholars have emphasized the characteristics of SLU from different perspectives, including (1) SLU requires that the interests of future generations are not harmed [13]; (2) SLU promotes simultaneous ecological, economic and social development [14]; and (3) SLU is a long-term cyclical business process [15].

The rapid development process in China has deteriorated the ecological environment and reduced land use efficiency [16, 17]. Therefore, the improvement of land use efficiency and the achievement of sustainable land development are of great practical importance. The coordination of land occupation, use, and dominance is important for achieving SLU [18]. The economic system and development model [19] and government policies [20] play important roles in this process. In terms of government policy, the current research has mainly focused on land administration systems [21] and land governance policies [22–29]. Although some scholars have studied SLU from the perspectives of poverty relief policy [30], renewable energy policy [31], and economic policy frameworks [32], the literature examining the impacts of other policies (those that indirectly affect land use) on SLU is inadequate. Therefore, the aim of this paper is to analyse how SLU in China have evolved under the influence of policies that indirectly affect land use. This also requires that the policy examined in this paper has had a major impact. In our study, we therefore use the example of China’s Carbon Emission Trading Scheme (CETS) policy.

The CETS is a classical policy with realistic implications, and it was officially implemented in 2013–2014 in six provincial pilot areas (Shanghai, Beijing, Tianjin, Guangdong, Hubei, and Chongqing). The CETS is a landmark exploration in China’s energy conservation and emission reduction policy, and it is the most effective and promising environmental regulation policy [33]. Nearly 3,000 heavily polluting enterprises in six pilot provinces were included in the scope of CETS regulation [34]. In the process of carbon regulation of polluting enterprises, the CETS helps enterprises to innovate and improve themselves, thus achieving the goals of reducing the marginal cost of output, reducing energy consumption per unit of output, and reducing emissions per unit of output. Indirectly, depending on the agglomeration characteristics of the pollution [35, 36], CETS improves not only the SLU of the region, but also the SLU of other regions. Based on the above analysis, it can be inferred that CETS is able to influence SLU. However, the literature discussing the effects of the CETS on SLU is scarce. Relevant studies have confirmed the effects of the CETS on land use [37, 38], but the element of "sustainability" has not been fully considered. At the same time, existing studies have been relatively subjective in the "selection of indicators" aspect of the SLU indicator evaluation system.

Our study designed a strategy based on the difference-in-difference (DID) method in order to construct a more objective evaluation system of SLU indicators, and in this way, it examined the impacts of the CETS on the evolution of SLU. The possible contributions of this study are (i) screening of the component indicators of SLU using the DID method, which enables the inference of causal relationships between variables. This will allow us to more objectively obtain the secondary indicators of SLU that produce changes under the influence of the CETS; and (ii) enriching the theory and method of CET and SLU.

The remainder of this paper is organized as follows: Section 2 introduces the research object, the design process of the data and the research methods; Section 3 reports the measurement process, the results and the spatio-temporal evolution of the SLU variables; Section 4 concludes the findings of the study and identifies the corresponding inspirations and shortcomings of the study.

## 2. Data and methods

### 2.1 Main study areas and data source

The study area of this paper is 30 provincial regions in China (excluding Hong Kong, Macao, Taiwan and Tibet). Among them, we mainly focus on 6 study regions, i.e., the pilot regions implementing CETS—Beijing, Tianjin, Shanghai, Hubei, Chongqing and Guangdong.

Among these pilot areas, Beijing and Shanghai are the political and financial centers of China, respectively, and they are significant to China’s economic development. Guangdong is the largest province in China in terms of GDP, and it has a large workforce.

Chongqing is located in western China, which is a nationally important advanced manufacturing center and financial center, as well as an internationally comprehensive area. Similarly, Hubei is an economic and educational base in central China.

The raw data were primarily gathered from the China Statistical Yearbook and the China Research Database Service Platform (https://www.cnrds.com/, accessed on Oct, 2022). The raw data on CO2 emissions were measured by Shan et al. [39, 40] and provided by the China Emission Accounts and Datasets (https://www.ceads.net/data/province/, accessed on Oct, 2022). We conducted this study using statistical data from 30 provincial-level regions in China from 2011 to 2017. The current literature typically refers to 2014 as the year in which the CETS produced shocks [41]. Since the CO_2_ emissions data were most recently updated for 2017, 2017 was taken as the end year of this study. Since the Five-Year Plans have greatly affected the development strategy of China, this study used the starting point of the twelfth Five-Year Plan as the initial year (2011) of this study.

### 2.2 Measurement strategy

**Fig 1:**
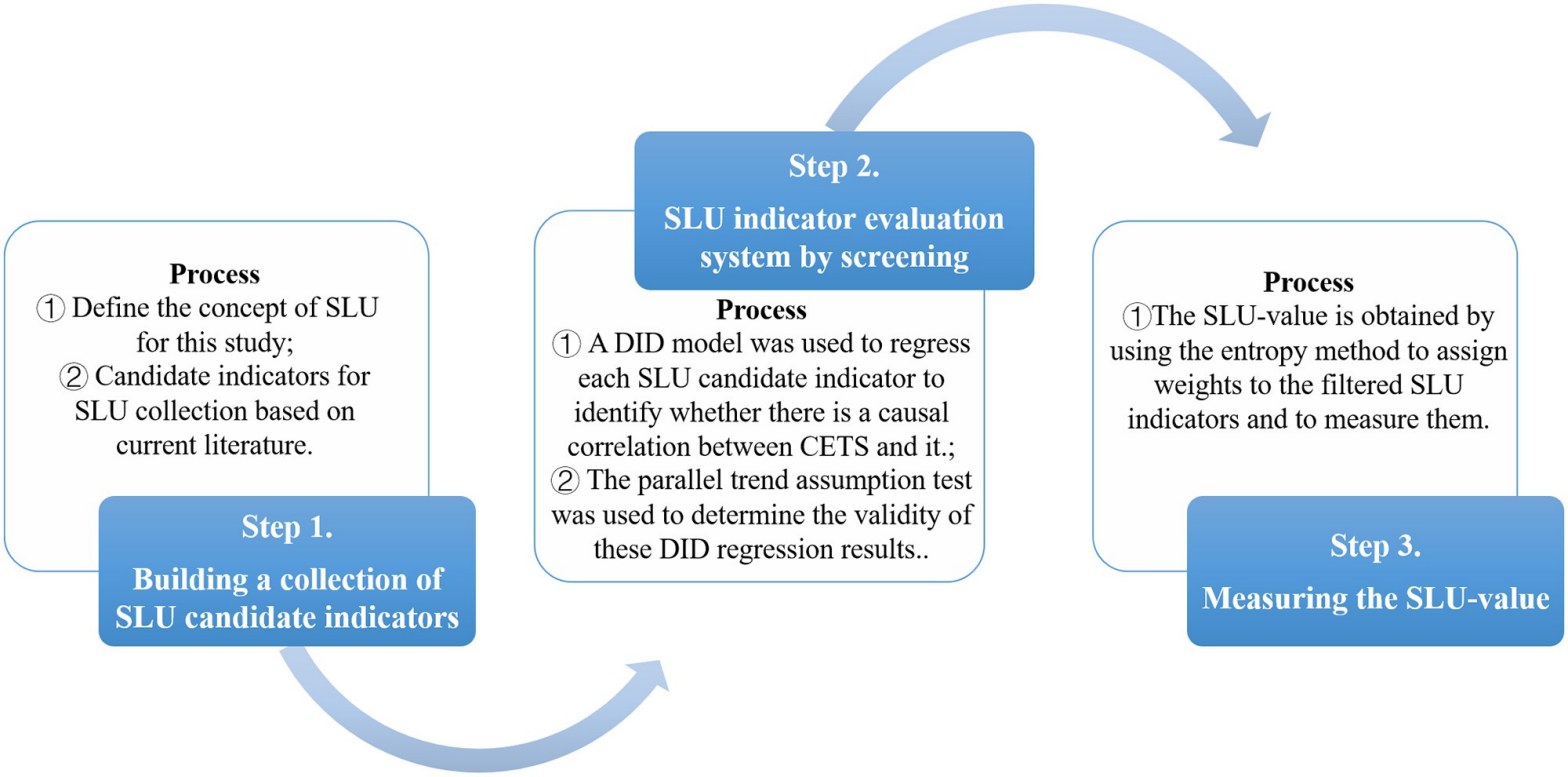
The process of the SLU measurements based on the DID estimation method.

In this subsection, we describe the indicator measurement strategy based on the DID estimation method mentioned above. Using this method, we obtained a more realistic picture of the changes in SLU under the influence of the CETS. Compared with traditional measurement strategies, this strategy yielded a more objective indicator evaluation system. Fig 1 provides an overview of this measurement strategy.

Step 1. According to the definition of SLU in Section 1 and the goals of this study, we measured the SLU values of the economic development and environmentally friendly progress dimensions, respectively. Since SLU in these two dimensions involves many indicators, it is impractical to list them all. We constructed a pool of candidate indicators on SLU. This was formed based on current literature on SLU measures. And then, the pool was used as the basis for the following screening work.

Step 2. We used the DID model to test the validity of each of the SLU candidates. Each SLU candidate collated in Step 1 was treated as an explained variable and subjected to regression analysis and a parallel trend assumption test. If the candidate indicator of SLU passed the significance level of at least 10% and met the parallel trend test, it meant that the implementation of the CETS did have an impact on the variable, i.e., the candidate indicator was determined to be a valid indicator. Otherwise, the indicator was deemed invalid and excluded from the SLU indicator evaluation system.

Step 3. Using the entropy method, the SLU indicator evaluation system (with the invalid indicators removed) obtained from the previous screening step was assigned weights to obtain the values of the composite SLU indicators.

### 2.3 Variable design

This subsection reports on the set of SLU candidate indicators, the explanatory variables, and the control variables. Section 2.3.1 discusses the content of Step 1 in Fig 1.

#### 2.3.1 Explained variable: SLU candidate indicators

This study uses the DID model to conduct a screening process for each SLU candidate indicator, so the explained variables are the individual SLU candidate indicators. according to the previous section, the SDGs consider three main dimensions of sustainable development as follows: economic development, environmentally friendly progress, and social progress [42, 43]. In combination with the SLU concept, the emphasis is on human production and living activities should be carried out within the carrying capacity of the land [44], such as environmental pollution. Based on this, and considering that China’s current land-based economic development is uncoordinated, unhealthy, and weak in sustainability, this study intended to construct an SLU index evaluation system from the perspectives of economic development and environmentally friendly progress.

The dimension of economic development emphasizes the use of a certain amount of land resources to obtain more economic benefits. This study built a candidate indicator set (Table 1) of SLU based on this dimension in terms of economic level, innovation capacity, and development rationality.

**Table 1:** Candidate indicators of SLU based on the economic development perspective.

Type	Description	Secondary Indicator	Unit	Sign
Economic level	Economic benefits from land use	GDP / land area	Billion Yuan / km^2^	ED1
	Economic benefits per capita from land use	GDP per capita of secondary and tertiary industries / area of built-up area of municipal districts	10,000 Yuan / km^2^	ED2
Innovation capacity	Local government attention to innovation development	Local public financial expenditure on S&T / land area	Million Yuan / km^2^	ED3
		Local public financial expenditure on S&T / resident population	Yuan / ten person	ED4
	Innovation level in the region	Number of patents granted / land area	Items / km^2^	ED5
		Number of patents granted / resident population	Items / 10,000 person	ED6
	Effort level of R&D personnel in the region	Full-time equivalent of R&D personnel / land area	Hundred person-years / km^2^	ED7
		Full-time equivalent of R&D personnel / resident population	person-years / hundred person	ED8
Development rationality	Rationalization of regional industrial structure	The proportion of tertiary output	%	ED9

1. Economic level. GDP is a most commonly used indicator of economic level. It is a good indicator that can reflect an economy’s level within a region [45]. A larger GDP value indicates that more wealth was created in that region over a certain period. In this study, we used "GDP / land area" to measure the economic benefits generated by a region using land. Considering the significant effect of population size on GDP [46], this study also used”GDP per capita of secondary and tertiary industries / area of built-up area of municipal districts” to reflect the per capita efficiency of wealth creation from non-agricultural production activities in a region. There were two reasons for excluding the primary industry: (i) cities are the main carriers of human life [47] and the main drivers of national economic development [48], but their construction land is primarily derived from agricultural and environmental land [49, 50]; therefore, the efficient and sustainable use of urban land is important for sustainable land use development; and (ii) China’s urban land is primarily used for secondary and tertiary industrial activities.

2. Innovation capacity. China is transitioning towards an innovation-driven economic development model. The past crude economic development model with factor inputs can no longer meet the current needs of China. There is no doubt that innovation has become the source of China’s economic development. Since science and technology (S&T) research and development is based on a large amount of research funding, this study introduced "local public financial expenditure on S&T / land area" and "local public financial expenditure on S&T / resident population" to reflect a local government’s attention to innovation development. At the same time, from the perspective of innovation outcomes, the indicators "number of patents granted / land area" and "number of patents granted / resident population” were chosen to reflect the level of innovation within a region [51]. Finally, research and development workers, as the carriers of innovation results, are obviously an important factor in measuring the innovation level, and so this paper also selected "full-time equivalent of R&D personnel / land area" and "full-time equivalent of R & D personnel / land area" as indicators [52].

3. Development rationality. For countries such as China, which were in the factor-input development mode in their early years, one of the important marks of economic quality improvement is the change in economic structure. This is reflected by a decrease in the share of output in a secondary industry and an increase in the share of output in a tertiary industry. Therefore, the indicator “the proportion of tertiary output” was used to reflect the rationality of SLU in a region with respect to economic activities.

The environmentally friendly progress dimension emphasizes reduced pollution emissions, more economic output with the same pollution emissions, and greening ecological construction. This study built a candidate indicator set (Table 2) of SLU based on this dimension in terms of pollution emissions intensity, economic benefits of pollution, and greening construction.

**Table 2:** Candidate indicators for SLU based on the environmentally friendly perspective.

Type	Description	Secondary Indicator	Unit	Sign
Pollution emission intensity	Industrial wastegas emissions	Industrial wastewater emissions / land area	Thousand standard cubic meters / km^2^	EF1
		Industrial wastewater emissions / resident population	Standard cubic meters / 10,000 people	EF2
	Industrial wastewater emissions	Industrial wastewater emissions / land area	Million tons / km^2^	EF3
		Industrial wastewater emissions / resident population	Tons / person	EF4
	Industrial soot emissions	Industrial wastewater emissions / land area	one hundred thousand tons / km^2^	EF5
		Industrial wastewater emissions / resident population	Hundred tons / person	EF6
	Industrial SO_2_ emissions	Industrial SO_2_ emissions / land area	Hundred tons / km^2^	EF7
		Industrial SO_2_ emissions / resident population	Tons / person	EF8
	CO_2_ emissions	CO_2_ emissions / land area	Tons / km^2^	EF9
		CO_2_ emissions / resident population	Tons / person	EF10
Economic benefits of pollution	Value of output per unit of energy consumption	Value added of secondary industry / industrial energy consumption	Yuan / KWh	EF11
Greening construction	Forest construction	Total area of forest / land area	%	EF12
		Total area of forest / resident population	Hectare / person	EF13
	Afforestation construction	Total area of afforestation / land area	%	EF14
		Total area of afforestation / resident population	Hectare / hundred person	EF15

1. Pollution emissions intensity. Firstly, the common industrial pollution emission indicators (industrial waste gas, industrial wastewater, industrial soot, and industrial SO_2_) were selected to reflect the pollution emissions from industrial production; secondly, CO_2_ emissions were selected to reflect the pollution from human production and operation, as well as that from living activities in the area; and thirdly, the above indicators were divided by "land area" and "resident population" to obtain the relative values of the indicators, and there were 10 candidate indicators identified.

2. Economic benefits of pollution. Economic benefits of pollution are reflected by the”value added of secondary industry / industrial energy consumption”. Since it is not possible to better correspond a certain type of pollutant to its corresponding economic output, industrial energy consumption was used here to indirectly reflect the pollution emissions. The value added by secondary industries/industrial energy consumption reflects the efficiency of energy use by industrial enterprises in a region, and there is a positive relationship between energy output and environmental pollution emissions. Therefore, this indicator can also indirectly reflect the idea of "more economic output with the same level of pollution emission".

3. Greening construction. Forestry carbon sinks, which refer to the participation in forestry resource trading through market-based approaches, thus generating additional economic benefits, includes both forest management carbon sinks and afforestation carbon sinks. Forest management carbon sinks target existing forests to promote forest growth and increase carbon sinks through forest management methods. A forestry carbon sink is a useful attempt under the "double-carbon" goals. Enterprises can purchase a carbon sink index to compensate for their own excess carbon emissions, which provides a convenient and long-lasting mechanism for the economic transformation of forestry carbon sinks. It also provides useful support for forestry development. Therefore, "total area of forest / land area", "total area of forest / resident population", "total area of afforestation / land area", and "total area of afforestation / resident population " were included in the SLU candidate indicators.

#### 2.3.2 Explanatory variable: CETS dummy variable

Since the DID model is used in this study, the explanatory variable is the dummy variable. The dummy variable consists of a time dummy (*Post*_*t*_) and an individual dummy (*Treat*_*i*_), reflecting whether a region is shocked by a policy (event) in a certain period. In this study, this variable then reflects whether the CETS policy is implemented in region i at year t. The specification is shown in Section 2.4.1.

#### 2.3.3 Control variable

The attention and investment of foreign companies bring advanced experience and technology to local companies, which is beneficial for local companies to improve their economic efficiency and operational capacity [53, 54]. Also, it helps firms to learn their pollution prevention strategies. Therefore, the foreign direction investment (FDI) variable is selected as the control variable in this paper to mitigate the impact on SLU from external side of China.

### 2.4 Methods

#### 2.4.1 DID method and parallel trend assumption test

The difference-in-difference (DID) model is one of the more popular econometric models, and it is often used in economics to assess whether there is a causal link between a shock generated by an event and an outcome. Further, DID naturally alleviates the endogeneity problem of econometric models. Therefore, this paper used DID to determine the causal relationship between each secondary indicator of SLU and the event shock (CETS) in order to determine the validity (causality) of the indicator. The basic form of the DID model applied to panel data is Eq (1):

$$Y_{i,t}=\alpha +\beta Treat_{i}\times Post_{t^{\prime }}+\delta Control_{i,t}+\mu_{i}+\gamma_{t}+\varepsilon_{i,t^{\prime }}$$ (1)

where *i* denotes the serial number of the province (1 ≤ *i* ≤ 30), *t* denotes the serial number of the year (2011 ≤ *i* ≤ 2017), *t’* is the time point of an event impact, *Y*_*i*,*t*_ is the explained variable, *Treat*_*i*_ × *Post*_*t’*_ is the key explanatory variable whose coefficient *β* is our interest value, *t’* is the first year of an event shock, *Control*_*i*,*t*_ represents the control variables, *μ*_*i*_ and *γ*_*t*_ are the spatial and time fixed effects, respectively, and *ε*_*i*,*t*_ is the error term.

The *Treat*_*i*_ × *Post*_*t’*_ is the CETS dummy variable in our study. Thus, when *Treat*_*i*_ × *Post*_*t’*_ = 1, it indicates that region *i* is a pilot province and the CETS has been implemented in year *t’*; otherwise, *Treat*_*i*_ × *Post*_*t’*_ = 0. Here, this paper takes 2014 as the initial year of CETS implementation. Eq (2) demonstrates this determination process.


$$Treat_{i}\times Post_{t^{\prime }}\left\{\begin{array}{ll} 1, & region\;i\;is\;the\;pilot\;area\;\&\;2014\leq t^{\prime }\leq 2017\\ 0, & otherwise \end{array}.\right.$$ (2)

If a secondary indicator passes at least a 10% significance test in the DID model, it initially indicates a causal relationship with the CETS. However, a parallel trend assumption is required to fully determine this. In this study, the parallel trend assumption was designed based on the event analysis framework strategy discussed below.

If the CETS has not yet been implemented, the treatment group and the control group have similar change trends; however, if the CETS has begun to be implemented, the treatment group and the control group show significant differences due to the impacts of the policy shock. Eq (3) is the regression equation used to achieve the above regression equation of the strategy:

$$SLU-value_{i,t}=\sum_{\tau =2011}^{\tau =2017}\beta_{\tau }Treat_{i}\times Dummy_{\tau }+\mu_{i}+\gamma_{t}+\varepsilon_{i,t^{\prime }}$$ (3)

where *Dummy*_*τ*_ is the time dummy variable. We were concerned with the significance of the coefficient *β*_*τ*_. In a later section, we plot the parallel trend map to better show the results of this process.

#### 2.4.2 Entropy method

The entropy method a more commonly used method of dimensionality reduction, which is based on the degree of variation between variables [4]. In our indicator measurement strategy, a set of valid indicators for SLU will be formed. In order to obtain a comprehensive SLU-value, we will use the entropy method to down-dimension the indicator set. Eqs (4)–(6) briefly describe this down-dimensioning process.


$$SLU_{i,t,p}=\frac{slu_{i,t,p}-\text{min}\left|slu_{i,t,p}\right|}{\text{max}\left|slu_{i,t,p}\right|-\text{min}\left|slu_{i,t,p}\right|}$$ (4)

$$\begin{array}{l} Info_{t,p}=-\text{ln}{\left(30\right)}^{-1}\sum_{i=1}^{n}\left(X_{i,t,p}\right)\text{ln}(X_{i,t,p})\\ X_{i,t,p}=\frac{SLU_{i,t,p}}{\sum_{i=1}^{30}SLU_{i,t,p}} \end{array}$$ (5)

$$Weight_{t,p}=\frac{1-Info_{t,p}}{K-\sum Info_{t,p}}$$ (6)

In Eq (4), we standardize the secondary indicators in the SLU indicator set to make their magnitudes consistent. Where *i* is the number of cross-sectional (0 < *i* ≤ 30), *t* means the year, *p* is the number of secondary indicator.

In Eq (5), we obtain the information entropy matrix *Info*_*t*,*p*_.

In Eq (6), we calculate the weights corresponding to each indicator in year t (*Weight*_*t*,*p*_). where *K* is the total number of secondary indicators in the SLU indicator sets.

## 3. Results

### 3.1 Validity analysis of the SLU candidate indicators

This subsection focuses on reporting the results of the screening of the SLU candidate indicators (Tables 3 and 4). We used StataSE-64 software to implement the regression analysis of Eq (2). An SLU candidate was considered a "valid SLU indicator" if it passed the significance test of at least 10% and met the parallel trend assumption test; otherwise, it was deemed an "invalid SLU indicator".

**Table 3:** Regression results for the SLU from the perspective of economic development.

Explained Variables	ED1	ED3	ED5	ED7
*Treat*_*i*_×*Post*_*2014*_	0.0293** (2.11)	0.3970** (2.40)	1.1089*** (2.85)	0.1201** (2.36)
Control variable	YES	YES	YES	YES
Fixed Effects (Year)	YES	YES	YES	YES
Fixed Effects (Provinces)	YES	YES	YES	YES
R^2^	0.1248	0.2404	0.2893	0.1900
Obs	210	210	210	210

Notes: t-value in parentheses.

***p < 0.01,

**p < 0.05.

**Table 4:** Regression results for the SLU from the perspective of environmentally friendly.

Explained Variables	EF8	EF10	EF11	EF12	EF14	EF15
*Treat*_*i*_×*Post*_*2014*_	-0.0057*** (-7.52)	-0.4143*** (-3.15)	0.7355* (1.98)	0.0154* (1.82)	0.0078* (1.92)	0.0073* (1.84)
Control variable	YES	YES	YES	YES	YES	YES
Fixed Effects (Year)	YES	YES	YES	YES	YES	YES
Fixed Effects (Provinces)	YES	YES	YES	YES	YES	YES
R^2^	0.2116	0.1835	0.1897	0.1400	0.1759	0.1421
Obs	210	210	210	210	210	210

Notes: t-value in parentheses.

***p < 0.01,

*p < 0.10.

Table 3 reports the regression results for the SLU candidate indicators from the perspective of economic development (Table 1). Due to the limited length of the table, Table 3 only shows the regression results for the SLU candidate indicators that passed the at least 10% significance level test. A total of four candidates—GDP/land area (sign ED1), local public financial expenditure on science and technology/land area (sign ED3), number of patents granted/land area (sign ED5), and full-time equivalent of research and development personnel/land area—met the basic requirements (sign ED7) and passed the test.

Table 4 reports the regression results for the SLU candidate indicators from the perspective of environmentally friendly progress (Table 2). Table 4 only shows the regression results for the SLU candidate indicators that passed the at least 10% significance level test. A total of six candidates—industrial SO2 emissions/resident population (sign EF8), CO2 emissions/resident population (sign EF10), value added by secondary industry/industrial energy consumption (sign EF1), total area of forest/land area (sign EF12), total area of afforestation/land area (sign EF14), and total area of afforestation/resident population—met the basic requirements (sign EF15) and passed the test.

**Fig 2:**
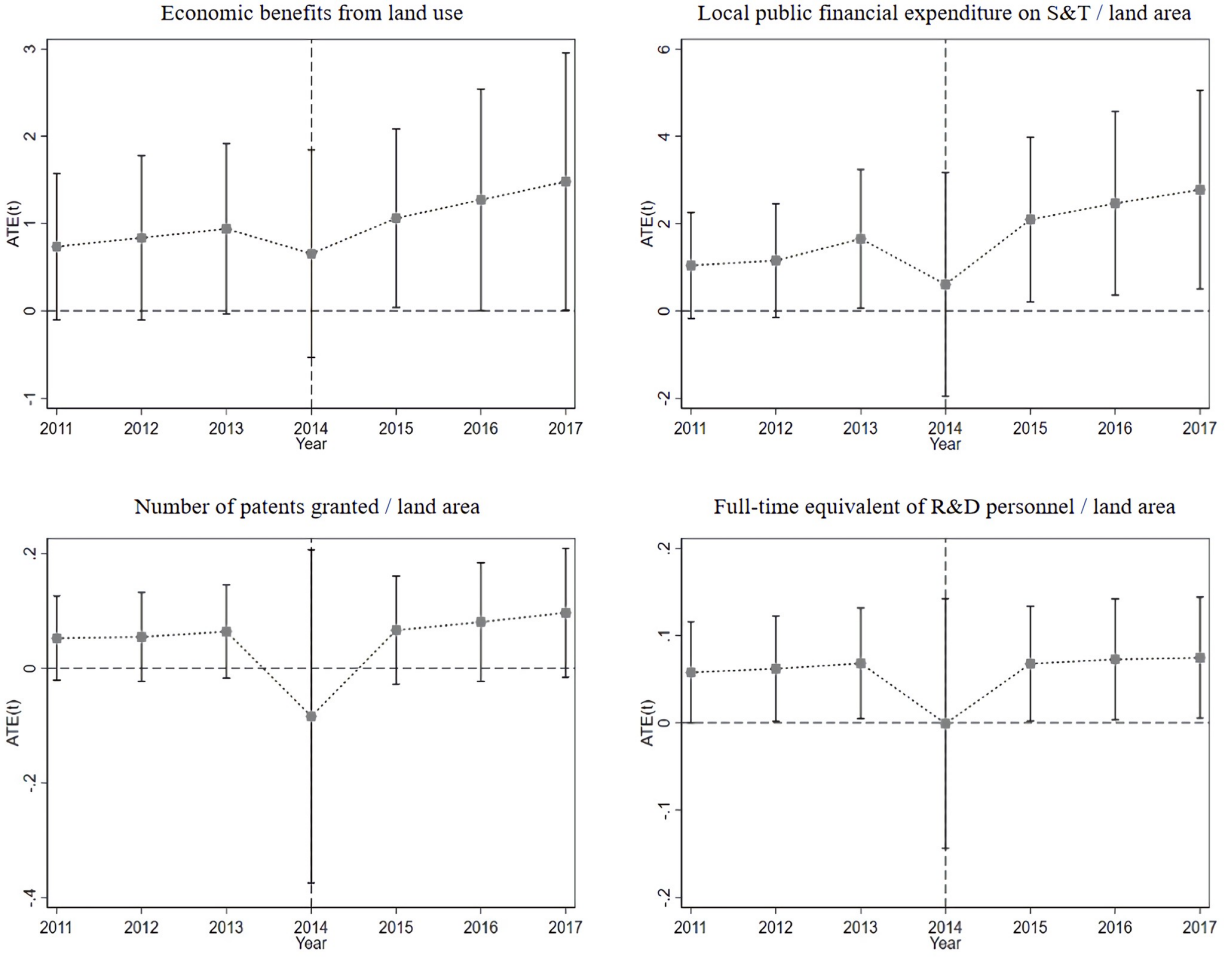
Parallel trend test for the SLU from the economic development dimension.

**Fig 3:**
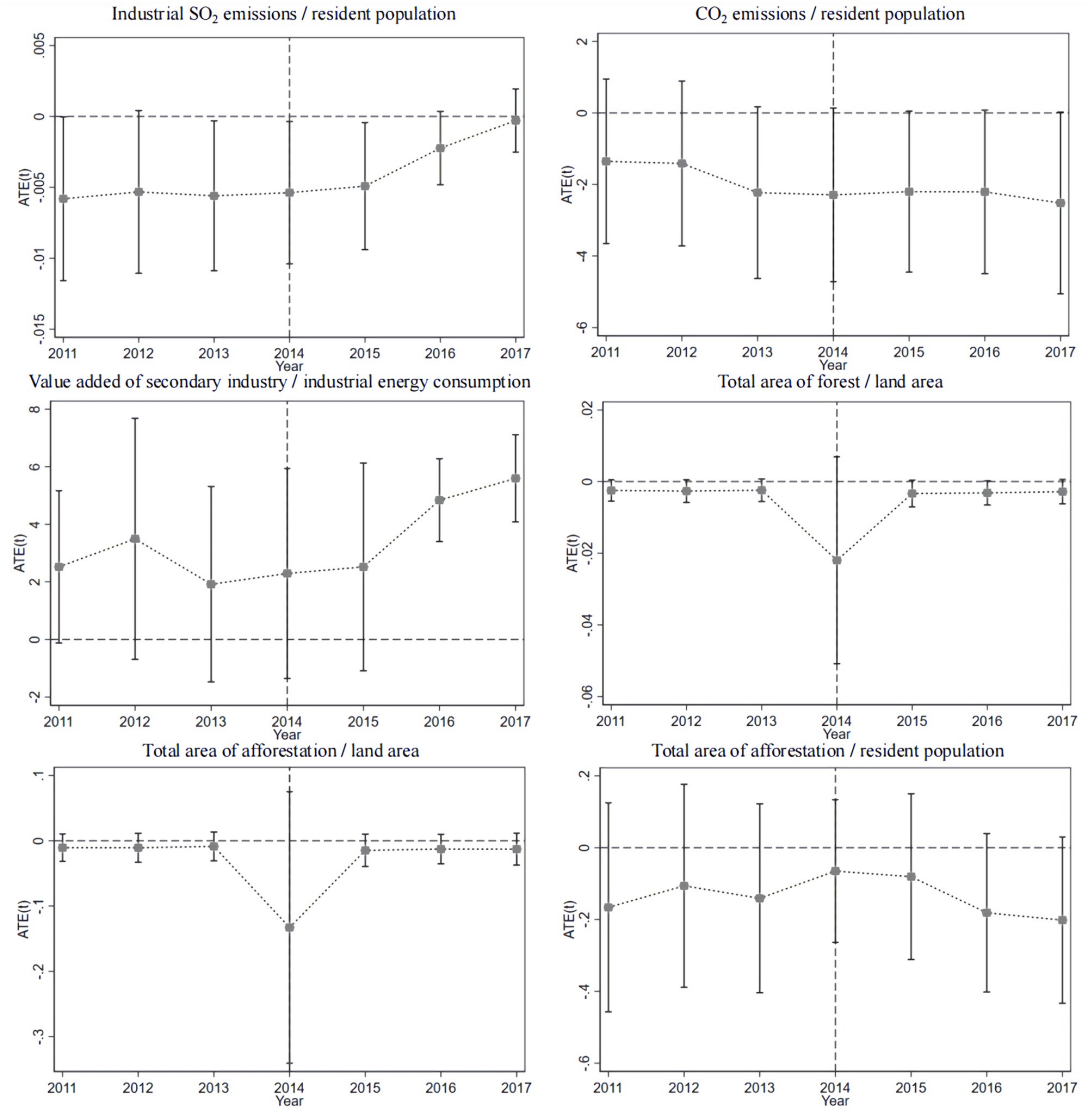
Parallel trend test for the SLU from the environmentally friendly dimension.

The regression results that passed the parallel trend assumption test were deemed to be valid results. For the SLU indicators that passed the first test—the significance test—the parallel trend assumption test was carried out, and we plotted Figs 2 and 3. It was clear that all the indicators in Tables 3 and 4 passed the test, indicating that the previous results were valid. It is worth mentioning that the magnitudes of the coefficients in Tables 3 and 4 were not informative for this study. We only examined whether they passed the significance test of at least 10%.

### 3.2 Measurement results of SLU

Using MATLAB R2021a, we measured the SLU indicators in Tables 1 and 2, respectively, i.e., two SLU indicators based on the economic development and environmentally friendly progress dimensions. Table 5 shows the weights of SLU secondary indicators based on economic development. From Table 5, we found that four of the nine candidate indicators were valid, and the indicators for economic level and innovation capacity passed the validity test while the indicators for development rationality were invalid.

**Table 5:** Weights of the SLU secondary indicators based on the economic development.

Year	ED1	ED3	ED5	ED7
2011	19.3523%	31.0172%	25.7841%	23.8463%
2012	19.3482%	31.1785%	25.7330%	23.7403%
2013	19.5692%	30.8334%	25.2474%	24.3500%
2014	28.2294%	22.5690%	23.4596%	25.7420%
2015	21.1645%	29.9028%	24.5804%	24.3523%
2016	20.8772%	30.5372%	24.3438%	24.2418%
2017	20.1453%	30.6908%	24.9523%	24.2115%

Notes: Please refer to Table 1 for the meaning of sign.

Table 6 shows the weights of the SLU secondary indicators based on environmentally friendly progress. As seen in Table 6, we found that 6 of the 15 indicators were valid, and all three levels of pollution emissions intensity, economic benefits of pollution, and greening construction passed the validity screening.

**Table 6:** Weights of the SLU secondary indicators based on the environmentally friendly.

Year	EF8	EF10	EF11	EF12	EF14	EF15
2011	17.0508%	8.5256%	17.3936%	13.0879%	16.1254%	27.8166%
2012	16.0004%	8.2325%	19.1207%	13.0454%	16.5184%	27.0826%
2013	17.3490%	9.1127%	20.1960%	11.9412%	15.2291%	26.1719%
2014	5.8290%	2.9848%	6.6634%	34.1706%	42.5873%	7.7648%
2015	14.3569%	7.1824%	17.3462%	17.5069%	26.6341%	16.9735%
2016	22.2718%	7.5170%	9.9213%	15.9056%	20.5707%	23.8136%
2017	33.1140%	8.2709%	8.3730%	9.7359%	15.6141%	24.8922%

Notes: Please refer to Table 2 for the meaning of sign.

### 3.3 Spatio-temporal evolution characteristics of SLU

#### 3.3.1 SLU based on economic development

Table 5 in the previous section shows the weight distribution of the secondary indicators based on the economic development dimension of SLU. The overall fluctuations in each indicator were not significant for the period 2011–2017, indicating that the variations in each indicator were relatively consistent.

The weight size can be used as a reference to indirectly distinguish the influenced degree of each indicator. As shown in Table 5, the weight of ED3 (local public financial expenditure on science and technology/land area) was 30% for a long time. The weight of ED5 (number of patents granted/land area) and ED7 (full-time equivalent of research and development personnel/land area) is more consistent and remains at 24% over time. The weight of ED1 (GDP/land area) was the smallest. This indicated that the CETS improved SLU primarily from the perspective of innovation capacity, and it had a weak impact on economic level and could not improve SLU from the perspective of development rationality.

The analyses of the specific indicators showed that local governments would strongly support innovation development because of the Chinese government’s performance race and its strong capacity to intervene in the market, and therefore, ED3 showed the highest fluctuations. Innovation results and the training and recruitment of research talents take time and are uncertain, and so the fluctuations in both were relatively weak. According to the previous analysis, the enactment of the CETS affected local economic development in the short term and promoted local economic development in the medium term and long term. The six pilot regions had already started to attempt innovation-driven transformation before that, and so their economies were less negatively affected, but they also had not yet formed obvious positive promotion effects. This was reflected in the weakest fluctuation in ED1. In addition, the four effective indicators were all "land area" indicators, which also indicated that the CETS had limited improvements in the SLU of the economic development dimension and had not yet produced a "per capita" level change.

In this paper, the implementation time of the policy was used as the boundary, setting 2011–2013 as the pre-implementation period and 2015–2017 as the post-implementation period, so as to take the average SLU value of the corresponding period, respectively. Meanwhile, the SLU value was divided into four categories: top five, top one-third, middle one-third, and the last one-third, and the corresponding numbers of the provincial areas were 5, 10, 10, and 10, respectively. We made Table 7 to show the spatio-temporal evolution of SLU from the economic development before and after the implementation of CETS by dividing the 30 provincial-level regions. The "Before" represents the rank of the average SLU from 2011 to 2013, while the "After" represents the rank of the average SLU from 2015 to 2017.

**Table 7:** Trends in SLU from the economic development before and after CETS.

East China			Central China			West China		
Rank	Before	After	Rank	Before	After	Rank	Before	After
Region			Region			Region		
Beijing^p^	++++	++++	Anhui	+++	+++	Chongqing^p^	++	++
Fujian	+++	+++	Henan	+++	++++	Gansu	+	+
Guangdong^p^	+++	+++	Heilongjiang	+	+	Guangxi	+	+
Hebei	++	++	Hubei^p^	++	++	Guizhou	+	+
Hainan	++	++	Hunan	++	++	Inner Mongolia	+	+
Jiangsu	++++	++++	Jilin	+	+	Ningxia	+	+
Liaoning	++	++	Jiangxi	++	++	Qinghai	+	+
Shandong	+++	+++	Shanxi	++	++	Sichuan	++	++
Shanghai^p^	++++	++++				Shaanxi	++	++
Tianjin*	++++	++++				Xinjiang	+	+
Zhejiang	++++	+++				Yunnan	+	+

Notes:

^++++^ refers to the top 5;

^+++^ refers to the top one-third;

^++^ refers to the middle one-third;

^+^ refers to the last one-third.

Pilot areas are marked with a "p" in the upper corner

Table 7 shows that before the implementation of the CETS (2011–2013), the "Top 5" provincial regions in terms of SLU were Beijing, Tianjin, Jiangsu, Shanghai, and Zhejiang. Beijing, Tianjin, and Shanghai were among the policy pilots, accounting for 60%. Among the pilot regions, Chongqing and Hubei were not ranked in the "top one-third".

Table 7 shows that after the implementation of the CETS (2015–2017), the "Top 5" provincial regions in terms of SLU ranking were Beijing, Tianjin, Shanghai, Henan, and Jiangsu. Beijing, Tianjin, and Shanghai were among the pilot regions, accounting for 60%, and Chongqing and Hubei were still not ranked in the "top one-third".

By region, the spatial distribution pattern of SLU based on the economic development dimension was very stable before and after the implementation of the policy. During the period 2011–2017, the characteristic of "eastern region > central region > western region" was largely maintained. This finding echoed the aforementioned observation that it takes time to develop innovation. In addition, it could be found that the CETS did not significantly improve the SLU of the central and western pilot regions (Hubei and Chongqing) in terms of innovation capacity, and it could also be concluded that the CETS had limited effectiveness on the economic development dimension.

#### 3.3.2 SLU based on environmentally friendly

Table 6 in the previous section shows the weight distributions of the secondary indicators of SLU based on the environmentally friendly progress dimension. The weights of some indicators changed significantly during the period 2011–2017, and the main turning point was 2014.

With the weight size as a reference, it was possible to indirectly distinguish the affected degree of each indicator. From Table 6, the weight of EF8 (industrial SO_2_ emissions/resident population) changed significantly, from 17% in 2011 to 33% in 2017,. The weight was stable from 2011 to 2013, and after dropping abruptly to a low of 5.8% in 2014, it began to increase year after year. The weight of EF10 (CO_2_ emissions/resident population) remained at approximately 8% overall, but similar to the case of EF8, it plunged to a low value of 2.98% in 2014 and then began to increase year after year, though not as much as EF8. Similarly, EF11 (value added by secondary industries/industrial energy consumption) and EF12 (total area of forest/land area) had significantly lower weights, while EF14 (total area of afforestation/land area) and EF15 (total area of afforestation/resident population) showed slight decreasing trends. From the perspective of the corresponding indicators, the CETS could improve SLU from the following three perspectives in the environmentally friendly progress dimension: pollution emission intensity, economic benefits of pollution, and greening construction. The impact of the CETS on the economic benefits of pollution was weak, while the impacts on pollution emission intensity and greening construction were comparable, which reflected the basic objective of the CETS as an environmental regulation policy.

The results of the analyses of the specific indicators showed the following: (1) The simultaneous fluctuations in EF8 and EF10 reflect the view that CO_2_ and SO_2_ pollution are certainly homogeneous, as described in the previous section. The significant decreases in the corresponding weights in 2014 were due to the significant reductions in emissions in the pilot areas with high pollutant emissions such that the emission levels in the pilot areas were closer to those in other areas, thus realizing significant decreases in the weights. The subsequent increases in the weights may have been due to the transfers of factories from the pilot regions to other regions. As mentioned earlier, factories cannot achieve a low-pollution production process in a short period of time, and they have to relocate if they do not want to stop working or leave the industry. This was also reflected in EF11. (2) EF12, EF14, and EF15 all reflect the development of forestry and forestry carbon sinks in China as a result of the CETS, and the decreasing weight of EF12, which is also known as forest cover, reflected the gradual decrease in the level of forest cover in each region. The decreasing weight of EF12 reflected the gradual approach of the forest cover level in each region. Similarly, the weights of EF14 and EF15, which reflected the area of afforestation, decreased slightly from year to year, reflecting a slight convergence in the area of afforestation in each region of China. Afforestation refers to artificial afforestation and aircraft afforestation, and planting is required on all lands that can be afforested, such as barren mountains and sand dunes. At the same time, after inspection and acceptance to meet the requirements of the afforestation technical regulations, a survival rate of 85% or more is required to be recognized as afforestation area. Therefore, it was more restrictive and showed a smaller change in weighting.

Using the same strategy, we made Table 8 to show the spatio-temporal evolution of SLU from the environmentally friendly.

**Table 8:** Trends in SLU from the environmentally friendly before and after CETS.

East China			Central China			West China		
Rank	Before	After	Rank	Before	After	Rank	Before	After
Region			Region			Region		
Beijing^p^	+	+++	Anhui	++++	++	Chongqing^p^	+++	++++
Fujian	+	+	Henan	++	++	Gansu	+	++
Guangdong^p^	++	+++	Heilongjiang	++	+++	Guangxi	++++	+++
Hebei	++	++++	Hubei^p^	++	+	Guizhou	++	++
Hainan	+++	+	Hunan	+	+	Inner Mongolia	++	++
Jiangsu	+	+	Jilin	++++	+	Ningxia	++++	++
Liaoning	+	++	Jiangxi	+	+	Qinghai	+	++
Shandong	+++	++++	Shanxi	+	+++	Sichuan	+	++++
Shanghai^p^	+++	+				Shaanxi	+++	+
Tianjin*	++	++++				Xinjiang	++	++
Zhejiang	++++	+				Yunnan	++	++

Notes:

^++++^ refers to the top 5;

^+++^ refers to the top one-third;

^++^ refers to the middle one-third;

^+^ refers to the last one-third.

Pilot areas are marked with a "p" in the upper corner

Table 8 shows that before the implementation of the CETS (2011–2013), the "Top 5" provincial regions in terms of SLU were Jilin, Anhui, Zhejiang, Guangxi, and Ningxia. Among the pilot regions, only Chongqing and Shanghai were in the "top one-third" group, while Beijing was in the "last one-third" group. There is no doubt that the SLU values (for the environmentally friendly progress dimension) of the pilot regions were low before the implementation of the CETS. There are two reasons for the low SLU values of Beijing: (1) Beijing has a more developed industry and is located in the north of China, which has a high demand for heating in the winter and, therefore, has higher CO_2_ and SO_2_ emissions; and (2) Beijing has more difficulties with afforestation due to its own geographical conditions. It is worth mentioning that China’s economic development model in the 2010s had not yet been transformed, and so the economic level was often negatively correlated with environmental pollution and greenery construction.

Table 8 shows that after the implementation of the CETS (2015–2017), the "Top 5" provincial areas in terms of SLU were Tianjin, Sichuan, Chongqing, Hebei, and Shandong. Tianjin and Chongqing were among the pilot areas, accounting for 40%. Hubei and Shanghai were downgraded, but Beijing, Tianjin, and Chongqing were upgraded while Guangdong maintained its original ranking. In addition, it could be found that the distribution of the SLU values of each group at that time had the following spatial clustering characteristics: (1) the northwestern region was in the "middle one-third"; (2) the Beijing-Tianjin-Hebei Urban Agglomeration and its surroundings, the Sichuan-Chongqing Urban Agglomeration, and the Guangdong were at least in the "top one-third"; and (3) the SLU values of the Pearl River Delta (PRD) Urban Agglomeration and its neighboring areas was generally downgraded. The reasons for the phenomena described above are as follows: (1) the improvement in the Beijing-Tianjin-Hebei Urban Agglomeration can be attributed to "double-carbon" policies such as the CETS. Beijing and other regions have been gradually implementing the "coal to gas" heating strategy and strengthening their implementation of work stoppage orders in autumn and winter. (2) The Sichuan-Chongqing Urban Agglomeration and Guangdong have abundant forestry resources. As a result of policies such as the CET, forestry carbon sinks have flourished in these regions, enabling them to rapidly increase their green construction levels. (3) As the Chinese forestry carbon sink market has gradually improved, enterprises can purchase carbon sinks to offset their emissions. As a result, various forms of carbon sink forests are gradually being built throughout the country, especially in the less economically developed regions with better ecological environments. In contrast, areas such as the PRD Urban Agglomeration, with its large population and expensive land, can hardly make up for the cost of developing carbon sink forests. In this process, the SLU values of the PRD urban agglomeration and its surrounding areas decreased significantly.

From a regional perspective, the spatial distribution pattern of SLU based on the environmentally friendly progress dimension evolved significantly before and after the policy implementation. During 2011 and 2017, it emerged from nothing to a spatial clustering feature with urban agglomerations as the core. At the same time, combined with the aforementioned analysis, we found that the improvement effect of the CETS on the SLU indicators for the environmentally friendly progress dimension was closely related to the locational factors (economic level, ecological environment, land area, etc.). Alternatively, the locational factors determined the upper limits of the optimization of SLU in the environmentally friendly progress dimension in the region.

## 4. Conclusions and inspirations

This study examined the optimal role of the CETS on SLU primarily from a geoeconomics perspective. This paper took 30 provincial-level regions (including 6 pilot areas) in China from 2011 to 2017 as the study subjects. Through an evaluation strategy based on the DID estimation method, this study constructed SLU indicator systems with economic development and environmentally friendly progress dimensions, respectively. Therefore, the strategy of this study emphasises the causal relationship between events and evolution as compared to existing methods of measuring indicators. With the help of the entropy method, this paper discussed the spatio-temporal evolution trend of SLU before and after the implementation of the CETS, and we reached the following conclusions: (1) The CETS can effectively improve SLU from the perspectives of economic development and environmentally friendly progress, and the impacts have largely been in the pilot areas. And, its effectiveness is closely linked to local locational factors. (2) For the dimension of economic development, the CETS has not changed the provincial distribution patterns of SLU at all, and they have remained "high to low, east to west". However, for the environmentally friendly progress dimension, the CETS has significantly changed the provincial distribution patterns of SLU, which are characterized by spatial agglomeration with urban agglomerations such as the PRD and the YRD as the core. (3) The screening results of the SLU indicators based on economic development showed that the CETS largely improved the innovation capacities of the pilot regions, though its impact on economic levels was relatively small. Similarly, the screening results of the SLU indicators based on environmentally friendly progress showed that CETS largely acted on reducing pollution emission intensity and strengthening greening construction, and they showed only short-term effects on improving energy use efficiency. Despite these findings, our study has the following limitations: (1) as CETS is a quasi-natural experiment, it is not possible to exclude all confounding factors (other policies, international influences, etc.) in the same way as in a natural experiment; (2) the candidate SLU indicators in this study are derived from the literature in the field of SLU. However, due to space constraints, it was not possible to test the validity of all indicators from the current literature; (3) we used the entropy method to measure the weights of each secondary SLU indicator. Future research could further investigate using other measures (SBM-DEA method, factor analysis method, etc.) to compare the impact of different measures on the results.

Based on the above conclusions, we obtained the following insights: (i) Although the CETS is an environmental regulation tool for CO_2_ pollutants, its policy effects are transmitted to many aspects due to the close connections between carbon emissions and human life and production and operation. Therefore, it should not be regarded as a mere emissions reduction policy. (ii) The implementation of the CETS is bound to harm some enterprises, especially small- and medium-sized energy-consuming enterprises. However, at the provincial level, the CETS did not cause economic setbacks in the pilot provinces, but rather, it improved the SLU levels of the region in terms of innovation capacity, pollution intensity reduction, and green construction. (iii) The formulation of environmental regulatory policies and the assessment of "double-carbon" performance for local governments must be appropriate to the local context, and they should be adapted to their own locational factors.
